# The stroke east Saxony pilot project for organized post‐stroke care: a case–control study

**DOI:** 10.1002/brb3.455

**Published:** 2016-04-28

**Authors:** Ulf Bodechtel, Kristian Barlinn, Uwe Helbig, Katrin Arnold, Timo Siepmann, Lars‐Peder Pallesen, Volker Puetz, Heinz Reichmann, Jochen Schmitt, Jessica Kepplinger

**Affiliations:** ^1^ Department of NeurologyUniversity Hospital Carl Gustav CarusTechnische Universität DresdenDresdenGermany; ^2^ Center for Evidence‐Based HealthcareUniversity Hospital Carl Gustav CarusTechnische Universität DresdenDresdenGermany

**Keywords:** Post‐stroke program, secondary stroke prevention

## Abstract

**Background:**

Low adherence to secondary prevention guidelines in stroke survivors may increase the risk for recurrent stroke and adversely impact quality of life. We aimed to determine the feasibility of a self‐developed standardized post‐stroke pathway and its impact on secondary stroke prevention and long‐term outcome in patients with acute stroke.

**Methods:**

Consecutive patients with acute stroke were prospectively included in a standardized post‐stroke pathway accomplished through a single certified CM (case manager), which comprised educational discussions and quarterly checkups for vascular risk factors and adherence to antithrombotic/anticoagulant medication in addition to usual care. At 12 months, we compared achieved target goals for secondary prevention, functional outcome, stroke recurrence, and vascular death with age‐ and gender‐matched controls that received only usual care after stroke.

**Results:**

We included 45 cases and 45 controls. The following target goals were more frequently achieved in CM‐patients than in controls: blood pressure (100% vs. 46.2%, *P* < 0.001), cholesterol (100% vs. 74.4%, *P* < 0.001), and body mass index (67.4% vs. 46.2%, *P* = 0.052). The CM‐intervention emerged as an independent predictor of favorable functional outcome (mRS ≤ 2) at 12 months after adjusting for stroke severity and systemic thrombolysis (OR: 4.27; 95%CI:1.2–15.21; *P* = 0.025). Quality of life was rated significantly higher in CM‐patients than in controls (*P* = 0.049). As opposed to controls, none of the cases experienced a recurrent stroke (0% vs. 13.3%; *P* = 0.026) or suffered from vascular death (0% vs. 6.7%; *P* = 0.242).

**Conclusions:**

Our pilot data suggest that organized post‐stroke care enhances achievement of secondary prevention goals. Its possible effect on stroke recurrence, long‐term disability, and quality of life is currently investigated in a prospective cohort study.

## Introduction

Stroke ranks as the fourth leading cause of death and constitutes a major contributor to long‐term adult disability in Western countries (Go et al. 2014). In the first year following stroke, recurrent events occur in 10–20% of patients, further increasing the degree of permanent disability and the risk of dying from a stroke (Chimowitz et al. 2011; Mohan et al. 2011). From an economic point of view, stroke ranges among the most expensive medical conditions adding up to direct costs of 7.1 billion Euro per year for stroke survivors in Germany alone without mentioning indirect costs related to compromised employability after stroke (Kolominsky‐Rabas et al. 2006).

In the recent years enormous efforts have been undertaken to fight the human and economic burden of stroke particularly by focusing on acute stroke management and rescue revascularization therapies (Broderick et al. 2013; Ciccone et al. 2013; Kidwell et al. 2013; Barlinn et al. 2014). On the other hand, an important element of the healthcare supply chain for stroke constitutes post‐stroke care. The strict implementation of goals for secondary prevention is crucial to overcome the high risk for long‐term disability and stroke recurrence. However, adherence to stroke prevention guidelines in stroke survivors is modest as recommendations are often not followed strictly by patients and primary care physicians (Röther et al. 2008; Bushnell et al. 2011). This may be due to a lack of patients’ education and advice with regard to risk factor management and life style changes as well as insufficient communication between the discharging hospital and primary care physicians (Bushnell et al. 2011). Organized post‐stroke care may help to enhance the dialog between the individual elements of the stroke's healthcare supply chain and improve adherence to secondary stroke prevention guidelines (Leistner et al. 2012). Recently established programs showed promising results for other diseases like diabetes mellitus and coronary heart disease, yet no such evidence‐based programs have been established for stroke survivors and current stroke guidelines are silent on this matter (Gaede et al. 2008; Giannuzzi et al. 2008; European Stroke Organisation (ESO) Executive Committee, ESO Writing Committee, 2008; Kernan et al. 2014).

Therefore, we developed a standardized and case management based pathway for post‐stroke care to ensure minimization of vascular risk factors, lifestyle changes, and adherence to secondary prevention medication. To guide a subsequent prospective cohort study, we aimed to determine the feasibility of this self‐developed post‐stroke pathway using a case–control design in patients with acute stroke and hypothesized that our coordinated post‐stroke care has a positive impact on secondary stroke prevention and long‐term functional outcome.

## Methods

### The SOS (stroke east Saxony) care project

From May 2011 to November 2011, a task force of stroke neurologists, primary care physicians, stroke nurses, ambulatory care nurses, physical therapists, and representatives of the German health insurance company “Allgemeine Ortskrankenkasse” (AOK Plus) developed a structured pathway for patients with acute cerebral ischemia and hemorrhagic stroke covering the first year after the index event. During the clinical phase of this 2‐year project (12/2011–05/2013), all pathway activities were carried out by a single licensed stroke case manager (U.H.) who was available on a regular basis (i.e., 40‐h per week except vacation time) and individually followed the participating patients over 1 year. A stroke expert physician was always available to discuss stroke‐related medical issues, whenever necessary.

**Figure 1:**
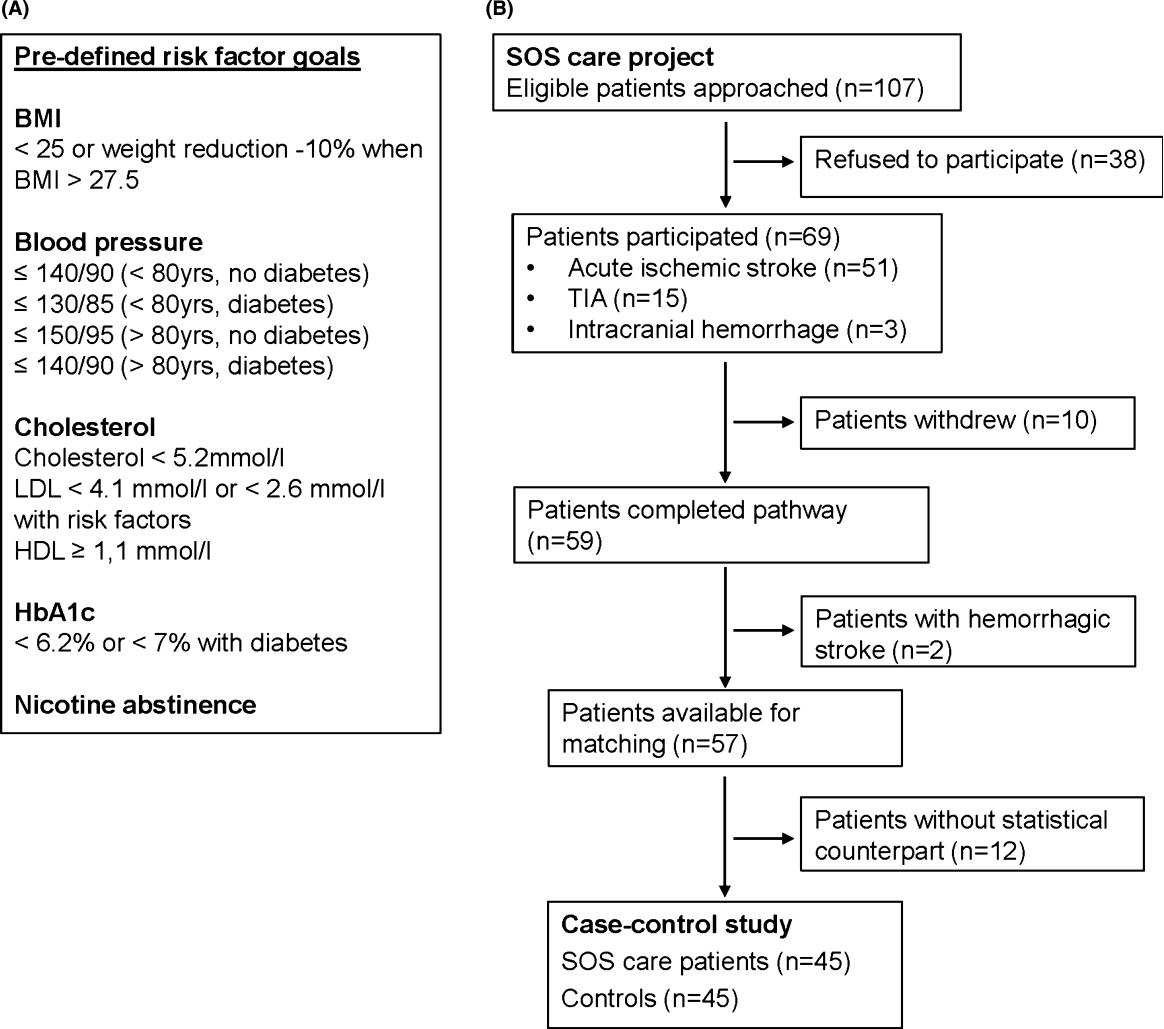
(A) Predefined risk factor goals. BMI indicates body mass index. (B) Study flowchart.

Consecutive acute stroke patients were prospectively approached by the case manager and invited to participate in the post‐stroke care program when following inclusion criteria were met: (1) acute ischemic or hemorrhagic stroke, or TIA (transient ischemic attack), (2) ongoing AOK Plus health insurance, and (3) residency in the city of Dresden, Germany. Exclusion criteria were pre‐stroke disability (defined as need of care for at least 3 h per day) and palliative care. Between day 2 and 3 of hospitalization, patients and if requested their proxies received a structured 30‐min educational discussion on stroke and its linkage to vascular risk factors, risk of recurrent stroke, and the importance of secondary prevention as well as healthy lifestyle changes (e.g., nicotine abstinence, weight reduction, healthy diet, physical activity). Within 1 week from hospital or rehab discharge, the case manager visited the patients at home to provide repeat stroke‐specific education (as described above) and to check whether secondary prevention medication complied with hospital discharge medication and the current European Stroke guidelines (European Stroke Organisation (ESO) Executive Committee, ESO Writing Committee, 2008). In addition, quarterly telephone contacts at 3, 6, and 9 months, and a final home visit at 12 months after the index stroke were conducted to check on vascular risk factors and achieved lifestyle changes. Target goals for vascular risk factors were predefined according to current recommendations given by the European Stroke guidelines (Fig. 1A) (European Stroke Organisation (ESO) Executive Committee, ESO Writing Committee, 2008). Blood samples for cholesterol and HbA1c were obtained by the primary care physician at 6 and 12 months, blood pressure and body weight measurements were performed quarterly by the patients themselves or the case manager. Whenever values on vascular risk factor deviated from the predefined goals the case manager informed the patients and their primary care physicians in order to consider appropriate changes in the treatment regimens. In addition to the scheduled visits, further personal or telephone contacts were conducted whenever felt necessary by the patient (e.g., ambulatory changes in the medication or medical status) or when the patient needed medical advice with regard to the stroke.

### Case–control study

To assess whether there is a signal of efficacy of our post‐stroke program, we retrospectively identified a comparison group from our institutional stroke database that consisted of patients with acute cerebral ischemia who were admitted between 01/2013 and 04/2013 (in order to ensure a 1‐year period after the index event), medically insured in any health insurance company except AOK Plus. All of these patients received usual care that was provided by primary care physicians after discharge from the acute hospital or rehabilitation, and was not standardized per study protocol. For reasons of comparability with the cases, we excluded control patients who had pre‐stroke mRS > 3 or palliative care after stroke. Usual care patients (controls) and SOS Care patients (cases) were individually matched for diagnosis according to International Classification of Disease (ICD‐10) coding (i.e., I60‐I64, G45), age (5‐year intervals), and sex by an independent assessor blinded to clinical data and the purpose of the study. All matched controls were appointed to our outpatient clinic or visited at home between 04/2014 and 06/2014 (approximately 12 months after the index stroke) for prospective assessments of cholesterol, HbA1c, blood pressure, body mass index, smoking habits, and secondary prevention medication and functional outcome using the mRS (modified Rankin scale).

### Patients’ satisfaction and quality of life

Patients’ satisfaction with post‐stroke care was assessed in cases and controls at the time of final follow‐up using a modified version of the ZUF‐8 questionnaire (Schmidt et al. 1989). The ZUF‐8 is a validated measurement instrument to evaluate patients’ general satisfaction with inpatient health care (Attkisson and Zwick 1982). The ZUF‐8 consists of eight items that cover various aspects of satisfaction on a 4‐point Likert scale. In order to address post‐stroke instead of inpatient care, we modified each ZUF‐8 item accordingly and extended the 8‐item questionnaire by two more items focusing on stroke diagnosis: (1) “I am aware of my personal stroke risk profile and I know how to control it”, and (2) “I understand the reason why several tests were performed during hospital stay”. Thus, the overall index score ranges between 8 and 40, with higher scores indicating higher satisfaction with care. In order to determine the reliability of the modified questionnaire we repeated the interview after 12 weeks to assess test–retest reliability.

Generic quality of life was assessed by the Euroqol 5D‐3L (EQ‐5D), a widely used measurement instrument that provides a simple descriptive profile and a single index value (visual analog scale) that can be used in the clinical evaluation of health care (Rabin and de Charro 2001).

### Outcome measures

We intended to compare achieved risk factor goals, functional independence (modified Rankin scale, mRS ≤ 1), favorable functional outcome (mRS ≤ 2) at 12 months as well as the need of institutional care, recurrent cerebral vascular events, and vascular death (defined as any death related to stroke or myocardial infarction) during the follow‐up period between cases and controls. We also intended to compare patients’ satisfaction and quality of life as assessed at 12 months between cases and controls. Patients with hemorrhagic stroke were not included in this case–control analysis due to low recruitment in the SOS Care project. The Dresden Institutional Review Board approved this study and written informed consent was obtained in all participants.

### Statistical analysis

Continuous and non‐continuous variables are presented as mean ± SD (standard deviation), median (interquartile range, IQR), and percentage as appropriate. Student *t* test, Wilcoxon rank‐sum test, chi‐square test, and Fisher's exact test were used to assess between group differences as appropriate. Univariate and multivariate logistic regression models were built to assess whether SOS Care was independently associated with outcomes of interest. All factors that emerged as predictor variables in the univariate analysis at *P* < 0.1 were included in the multivariate model as candidate variables and then removed by backward stepwise selection procedure with removal set at *P* = 0.2. Cohen's weighted *κ* was used to assess test–retest reliability for the modified ZUF‐8 with *κ *≥ 0.81, indicating very good/excellent; 0.61 ≤ *κ* < 0.81, good; 0.41 ≤ *κ* < 0.60, fair; and *κ* < 0.41, slight/poor agreement (Byrt 1996). Internal consistency of the modified ZUF‐8 was assessed by Cronbach's alpha with *α* > 0.9 indicating being excellent, *α* > 0.8 good, *α* > 0.7 acceptable, *α* > 0.6 questionable, *α* > 0.5 poor, and *α* < 0.5 unacceptable internal consistency (George and Mallery 2003). Significance level was set at *P* < 0.05. OR (Odds ratios) are presented with corresponding 95% confidence intervals (CI). The statistical software package STATA (Version 12.1, StataCorp., College Station, TX) was used for statistical analysis.

## Results

Of 107 stroke patients who met inclusion criteria, 69 (64.5%) agreed to participate in the 1‐year integrated stroke care pathway: 58% were male, mean age was 70.8 ± 14.2 years, median NIHSS (National Institutes of Health Stroke Scale) score was 2 (IQR, 0–5) points. Fifty‐one (73.9%) patients had acute ischemic stroke, three (4.4%) intracranial hemorrhage, and 15 (21.7%) TIA. During the follow‐up period, 10 patients (14.5%) withdrew from the program due to lack of motivation or moving away and two patients with intracranial hemorrhages were excluded. Of the remaining 57 patients, 12 were not found to have a statistical counterpart according to our predefined matching criteria leaving 45 cases and 45 controls for the final analysis (Fig. 1B).

Except for stroke severity, no significant baseline imbalances were present between the cases and controls (Table 1). There was no difference between cases and controls with regard to favorable functional outcome (mRS ≤ 2) at the time of discharge (44.4% vs. 57.8%; *P* = 0.206). At the program's closing visit, two SOS Care patients and six control patients were deceased at this time point. The mean follow‐up period for cases and controls was 12.2 ± 0.7 months and 14.0 ± 1.5 months, respectively**.**

**Table 1:** Baseline characteristics of SOS Care and control patients

Variable	Cases (*n* = 45)	Controls (*n* = 45)	*P* value
Male, *n* (%)	26 (57.8)	26 (57.8)	1.0
Age, mean ± SD	70.4 ± 14.1	70.5 ± 13.6	0.939
Body mass index, median (IQR)	26 (24–28)	25 (24–29)	0.825
Acute ischemic stroke, *n* (%)	37 (82.2)	37 (82.2)	1.0
NIHSS, median (IQR)	2 (1–5)	5 (1–12)	0.029
Previous Stroke/TIA, *n* (%)	5 (11.1)	9 (20.0)	0.384^a^
Systemic thrombolysis, *n* (%)	10 (22.2)	10 (22.2)	1.0^a^
Vascular risk factors, *n* (%)			
Arterial hypertension	41 (91.1)	45 (100)	0.117^a^
Hypercholesterolemia	38 (84.4)	39 (86.7)	1.0^a^
Nicotine	13 (28.9)	8 (17.8)	0.319^a^
Diabetes mellitus	14 (31.1)	14 (31.1)	1.0
Coronary artery disease	4 (8.9)	12 (26.7)	0.051^a^
Atrial fibrillation	8 (17.8)	13 (28.9)	0.319^a^

IQR, interquartile range; NIHSS, National Institutes of Health Stroke Scale; TIA, transient ischemic attack.

a Fisher's Exact test.

The following target goals were more frequently achieved in SOS Care patients than in controls: blood pressure (100% vs. 46.2%, *P* < 0.001), cholesterol (100% vs. 74.4%, *P* < 0.001), and body mass index (67.4% vs. 46.2%, *P* = 0.052). Further results on achieved vascular risk factors goals and medication adherence are detailed in Table 2. More patients in the case management group were functionally independent at 12 months after the index event than patients receiving only usual care (75.6% vs. 48.9%; *P* = 0.009). In an unadjusted logistic regression model with the controls as the reference group, case management emerged as significant predictor of functional independence at 12 months (OR: 3.23; 95%CI: 1.32–7.92; *P* = 0.010). Once baseline stroke severity and systemic thrombolysis were adjusted for in the model, this association no longer remained significant (OR: 1.62; 95%CI: 0.56–4.66; *P* = 0.369). However, case management appeared a strong independent predictor of favorable functional outcome (mRS ≤ 2) at 12 months after adjusting for baseline stroke severity and systemic thrombolysis (OR: 4.27; 95%CI: 1.2–15.21; *P* = 0.025). Less SOS Care patients needed institutional care than control patients (0% vs. 8.9%; *P* = 0.04). As opposed to controls, none of the cases experienced a recurrent stroke (0% vs. 13.3%; *P* = 0.026) or suffered from vascular death (0% vs. 6.7%; *P* = 0.242) during the follow‐up period.

**Table 2:** Achieved vascular risk factors goals and antiplatelet/anticoagulant medication adherence at 12‐month closing visit

Achieved goals, *n* (%)	Cases (*n* = 43)	Controls (*n* = 39)	*P* value
Body mass index	29 (67.4)	18 (46.2)	0.052
Blood pressure	43 (100)	18 (46.2)	<0.001^a^
Cholesterol	43 (100)	29 (74.4)	<0.001^a^
Nicotine abstinence	38 (88.4)	37 (94.9)	0.436^a^
HbA1c	39 (90.7)	34 (87.2)	0.730^a^
Antiplatelet/anticoagulant adherence	40 (93.0)	35 (89.7)	0.703^a^

a Fisher's Exact test.

### Patients’ satisfaction and quality of life

The ZUF‐8 response rate was 95.6% (*n* = 43) for cases and 86.7% (*n* = 39) for controls. According to the modified ZUF‐8, patients in the SOS Care group were significantly more satisfied with post‐stroke care than controls (median: 39 [IQR, 5] vs. 21 [15]; *P* < 0.0001). The test–retest reliability for the modified ZUF‐8 questionnaire was good for eight and fair for two items. Internal consistency of the modified ZUF‐8 was excellent (Cronbach's *α* 0.97).

The response rate for the EQ5D was 93.3% (*n* = 42) for cases and 86.7% (*n* = 39) for controls. Quality of life measured by the EQ5D questionnaire was rated significantly higher in SOS Care patients than in controls (median index: 0.887 [IQR, 0.099] vs. 0.788 [0.0625]; *P* = 0.049). The EQ visual analog scale also revealed a significant difference among groups in favor of the cases (median score: 80 [IQR, 20] vs. 60 [30]; *P* = 0.013).

## Discussion

Our pilot study showed that organized post‐stroke care accomplished through a single case manager was feasible and potentially effective in the first year following acute stroke. Given that most patients who completed our post‐stroke program achieved predefined secondary prevention goals, these findings suggest that the coordinated post‐stroke programs may aid in achieving guideline‐recommended target goals for vascular risk factors and lifestyle changes to prevent stroke recurrence.

To date, substantial amounts have been invested in acute stroke research yet only a few therapies have been proven effective in reducing long‐term disability and death from stroke (The National Institute of Neurological Disorders and Stroke rt‐PA Stroke Study Group, 1995; Vahedi et al. 2007; Hacke et al. 2008; Ringelstein et al. 2013; Jüttler et al. 2014; Berkhemer et al. 2015). Merely, a minority of stroke patients has access to and even fewer eventually benefit from acute stroke therapies (Adeoye et al. 2014). Consequently, growing awareness has been shifted toward the postacute phase of stroke, which has been recognized as pivotal target to improve medical and economic aspects of stroke care (Alvarez‐Sabin et al. 2009; Philp et al. 2013). However, following hospitalization or rehab discharge, most stroke patients are subsequently treated by primary care physicians seldom formally trained in the treatment of stroke or familiar with secondary stroke prevention guidelines. The Adherence eValuation After Ischemic stroke‐Longitudinal (AVAIL) registry, for instance, revealed that 1 year after the index event, only two‐thirds of stroke patients continued secondary preventive medication as prescribed at hospital discharge and discontinuation of medication mostly followed the recommendation of primary care physicians (Bushnell et al. 2011). The fact that 12 months adherence to evidence‐based secondary preventive medication was highest among those who had a prompt appointment with a physician, were satisfied with patient–physician communication and understood why medication was necessary emphasizes the importance of sufficient information provision to patients and sustained stroke expertise availability after hospital discharge. This is underpinned by a recent observation that among acute stroke patients who received written information on secondary prevention and healthy lifestyle during hospitalization, the majority was not able to reproduce this information sufficiently postdischarge (Lawrence et al. 2010). Our post‐stroke program embedded broad educational discussions twice – during the acute and the postdischarge phase – with an intended involvement of family members that seems particularly essential in patients with restricted ability to understand (e.g., due to aphasia). Involving proxy might thus facilitate patients’ reception of secondary stroke prevention information. In addition, patients were free to contact the case manager at any time during working hours in order to discuss stroke‐related issues further improving post‐stroke education of patients. The Global Stroke Community Advisory Panel recently presented a post stroke checklist for patients discharged and to be completed by the treating primary care physician (Ward et al. 2014). Although this approach was feasible and deemed helpful in organizing care, it does not substitute the know‐how of a certified and trained stroke case manager as appointed in our program. On the other hand, a large number of patients refused to participate in the program from the beginning mostly due to unspecific reasons, for example, no personal need for intensified care, refusal of home visits through the case manager. As a consequence informational discussions need to be further intensified by the case manager in order to promote awareness for secondary stroke prevention.

To the best of our knowledge, this is the first study that prospectively investigates a post‐stroke care program accomplished through a certified case manager with scheduled and nonscheduled appointments. In contrast, other prevention programs were exclusively telephone‐based or relied on the patients’ attendance to outpatient appointments (Leistner et al. 2012; Irewall et al. 2014; McAlister et al. 2014). A recent study comparing usual outpatient care with an organized post‐stroke program in patients with minor stroke and TIA showed that about 25% of patients who initially agreed to participate did not attend the scheduled outpatient follow‐up appointments (Leistner et al. 2012). Such low outpatient visit adherence may be overcome by complementary contacts initiated by a case manager with a 12‐month final home visit as deployed in our prevention program resulting in a follow‐up attendance of 85%. Nonetheless, considerable efforts were necessary during our 1‐year program to accomplish such attendance rate.

As opposed to other post‐stroke services, essential content of our program was the achievement of vascular risk factor goals proven effective in secondary stroke prevention and recommended by most stroke guidelines (European Stroke Organisation (ESO) Executive Committee, ESO Writing Committee, 2008; Kernan et al. 2014; Irewall et al. 2014; Andersen et al. 2000; Claiborne 2006). In our case–control analysis, all patients receiving SOS Care achieved target goals for blood pressure and cholesterol levels at the closing visit whereas only a half to two‐thirds of control patients fulfilled predefined risk factor targets. We speculate that these improved success rates for two of the most important modifiable risk factors for ischemic stroke may at least partly result from the individual interventions provided by the case manager. Two recent stroke prevention programs providing scheduled outpatient appointments in the first 6 months after the index event resulted in slightly lower success rates for blood pressure and cholesterol risk factor control (Leistner et al. 2012; McAlister et al. 2014). Our pilot results, therefore, allow the generation of the hypothesis that combinatory regular and off‐schedule individual contacts are superior to solely prescheduled appointments. Also, our results reflect case management guidance for 12 months and this prolonged follow‐up may further contribute to risk factor control in long term. The unusual high antiplatelet and anticoagulant medication adherence rate in the control group precluded any conclusion on the effect of the interventions made in the SOS Care patients. It cannot be determined whether this was because of an anomaly in the control group or a lack of effect of the intervention. However, as compared with adherence rates reported in the literature, our achieved rates appear much higher (Bushnell et al. 2011; Leistner et al. 2012). Lastly, levels of patients’ satisfaction and quality of life – both increasingly recognized as important patient‐reported outcomes in acute stroke trials – were significantly higher in those receiving organized post‐stroke than usual care and therefore may have contributed to overall sufficient risk factor control in our study patients as previously suggested by the AVAIL registry (Bushnell et al. 2011). Although our positive results on functional outcome, stroke recurrence, and vascular death point to a signal of efficacy of case management‐based post‐stroke care, it needs to be acknowledged that our study design does not allow definite conclusions in this matter and therefore should be validated in larger prospective trials.

Our study has limitations. First, our results are limited by the small number of cases. However, this study presents results of a pilot phase with limited funding available and only one single case manager was deployed in our program. Although this case manager run the program on a full‐time basis, the maximum number of includable patients was restricted by the manager's overall capability. Second, endpoints were not compared at equal time points since SOS care patients were enrolled prospectively, whereas controls were selected according to our stroke database and eventually followed up later than cases. Besides, lack of standardization of usual care may have introduced bias to the results. Third, we cannot provide data on patients who refused to participate in the program, but it can be assumed that motivated patients have completed the pathway introducing bias toward the intervention group. Fourth, the NIHSS at admission differed between groups; however, although the SOS Care group was less severely affected at baseline no differences with regard to discharge functional status were apparent between cases and controls.

The strengths of our study include the prospective design of the post‐stroke pathway with strict implementation of evidence‐based recommendations of current stroke guidelines, the overall high availability of a single case manager, the prospective assessment of variables of interest in control patients and the high follow‐up attendance rate for cases and control patients. Additionally, our study generated pilot data for sample size calculations for follow‐up studies with larger populations to confirm our findings and assess their generalizability in a multicenter approach with a projected sample size of 100 patients per group.

## Conclusions

Our pilot data suggest that organized post‐stroke care enhances achievement of secondary prevention goals. Its possible effect on stroke recurrence, long‐term disability, and quality of life is currently investigated in a prospective cohort study.

## Conflict of Interest

The authors declare that they have no conflict of interest.

## References

[brb3455-bib-0001] Adeoye, O. , K. C. Albright , B. G. Carr , C. Wolff , M. T. Mullen , T. Abruzzo , et al. 2014 Geographic access to acute stroke care in the United States. Stroke 45:3019–3024. 25158773 10.1161/STROKEAHA.114.006293 PMC5877807

[brb3455-bib-0002] Alvarez‐Sabin, J. , M. Quintana , M. A. Hernandez‐Presa , C. Alvarez , J. Chaves , and M. Ribo . 2009 Therapeutic interventions and success in risk factor control for secondary prevention of stroke. J. Stroke Cerebrovasc. Dis. 18:460–465. 19900649 10.1016/j.jstrokecerebrovasdis.2009.01.014

[brb3455-bib-0003] Andersen, H. E. , K. Schultz‐Larsen , S. Kreiner , B. H. Forchhammer , K. Eriksen , and A. Brown . 2000 Can readmission after stroke be prevented? Results of a randomized clinical study: a postdischarge follow‐up service for stroke survivors. Stroke 31:1038–1045. 10797163 10.1161/01.str.31.5.1038

[brb3455-bib-0004] Attkisson, C. C. , and R. Zwick . 1982 The client satisfaction questionnaire. Psychometric properties and correlations with service utilization and psychotherapy outcome. Eval. Program. Plann. 5:233–237. 10259963 10.1016/0149-7189(82)90074-x

[brb3455-bib-0005] Barlinn, K. , G. Tsivgoulis , A. D. Barreto , J. Alleman , C. A. Molina , R. Mikulik , et al. 2014 Outcomes following sonothrombolysis in severe acute ischemic stroke: subgroup analysis of the CLOTBUST trial. Int. J. Stroke 9:1006–1010. 25079049 10.1111/ijs.12340 PMC4227933

[brb3455-bib-0006] Berkhemer, O. A. , P. S. Fransen , D. Beumer , L. A. van den Berg , H. F. Lingsma , A. J. Yoo , et al. 2015 A randomized trial of intraarterial treatment for acute ischemic stroke. N. Engl. J. Med. 372:11–20. 25517348 10.1056/NEJMoa1411587

[brb3455-bib-0007] Broderick, J. P. , Y. Y. Palesch , A. M. Demchuk , S. D. Yeatts , P. Khatri , M. D. Hill , et al. 2013 Endovascular therapy after intravenous t‐PA versus t‐PA alone for stroke. N. Engl. J. Med. 368:893–903. 23390923 10.1056/NEJMoa1214300 PMC3651875

[brb3455-bib-0008] Bushnell, C. D. , D. M. Olson , X. Zhao , W. Pan , L. O. Zimmer , L. B. Goldstein , et al. 2011 Secondary preventive medication persistence and adherence 1 year after stroke. Neurology 77:1182–1190. 21900638 10.1212/WNL.0b013e31822f0423 PMC3265047

[brb3455-bib-0009] Byrt, T. 1996 How good is that agreement? Epidemiology 7:561. 8862998 10.1097/00001648-199609000-00030

[brb3455-bib-0010] Chimowitz, M. I. , M. J. Lynn , C. P. Derdeyn , T. N. Turan , D. Fiorella , B. F. Lane , et al. 2011 Stenting versus aggressive medical therapy for intracranial arterial stenosis. N. Engl. J. Med. 365:993–1003. 21899409 10.1056/NEJMoa1105335 PMC3552515

[brb3455-bib-0011] Ciccone, A. , L. Valvassori , M. Nichelatti , A. Sgoifo , M. Ponzio , R. Sterzi , et al. 2013 Endovascular treatment for acute ischemic stroke. N. Engl. J. Med. 368:904–913. 23387822 10.1056/NEJMoa1213701 PMC3708480

[brb3455-bib-0012] Claiborne, N. 2006 Effectiveness of a care coordination model for stroke survivors: a randomized study. Health Soc. Work 31:87–96. 16776026 10.1093/hsw/31.2.87

[brb3455-bib-0013] European Stroke Organisation (ESO) Executive Committee, ESO Writing Committee . 2008 Guidelines for management of ischaemic stroke and transient ischaemic attack 2008. Cerebrovasc. Dis. 25:457–507. 18477843 10.1159/000131083

[brb3455-bib-0014] Gaede, P. , H. Lund‐Andersen , H. H. Parving , and O. Pedersen . 2008 Effect of a multifactorial intervention on mortality in type 2 diabetes. N. Engl. J. Med. 358:580–591. 18256393 10.1056/NEJMoa0706245

[brb3455-bib-0015] George, D. , and P. Mallery . 2003 SPSS for Windows step by step: a simple guide and reference. 11.0 update, 4th ed. Allyn & Bacon, Boston, MA.

[brb3455-bib-0016] Giannuzzi, P. , P. L. Temporelli , R. Marchioli , A. P. Maggioni , G. Balestroni , V. Ceci , et al. 2008 Global secondary prevention strategies to limit event recurrence after myocardial infarction: results of the GOSPEL study, a multicenter, randomized controlled trial from the Italian Cardiac Rehabilitation Network. Arch. Intern. Med. 168:2194–2204. 19001195 10.1001/archinte.168.20.2194

[brb3455-bib-0017] Go, A. S. , D. Mozaffarian , V. L. Roger , E. J. Benjamin , J. D. Berry , M. J. Blaha , et al. 2014 Heart disease and stroke statistics–2014 update: a report from the American Heart Association. Circulation 129:e28–e292. 24352519 10.1161/01.cir.0000441139.02102.80 PMC5408159

[brb3455-bib-0018] Hacke, W. , M. Kaste , E. Bluhmki , M. Brozman , A. Dávalos , D. Guidetti , et al. 2008 Thrombolysis with alteplase 3 to 4.5 hours after acute ischemic stroke. N. Engl. J. Med. 359:1317–1329. 18815396 10.1056/NEJMoa0804656

[brb3455-bib-0019] Irewall, A. L. , L. Bergström , J. Ogren , K. Laurell , L. Söderström , and T. Mooe . 2014 Implementation of telephone‐based secondary preventive intervention after stroke and transient ischemic attack – participation rate, reasons for nonparticipation and one‐year mortality. Cerebrovasc. Dis. Extra 4:28–39. 24715896 10.1159/000358121 PMC3975210

[brb3455-bib-0020] Jüttler, E. , A. Unterberg , J. Woitzik , J. Bösel , H. Amiri , O. W. Sakowitz , et al. 2014 Hemicraniectomy in older patients with extensive middle‐cerebral‐artery stroke. N. Engl. J. Med. 370:1091–1100. 24645942 10.1056/NEJMoa1311367

[brb3455-bib-0021] Kernan, W. N. , B. Ovbiagele , H. R. Black , D. M. Bravata , M. I. Chimowitz , M. D. Ezekowitz , et al. 2014 Guidelines for the prevention of stroke in patients with stroke and transient ischemic attack: a guideline for healthcare professionals from the American Heart Association/American Stroke Association. Stroke 45:2160–2236. 24788967 10.1161/STR.0000000000000024

[brb3455-bib-0022] Kidwell, C. S. , R. Jahan , J. Gornbein , J. R. Alger , V. Nenov , Z. Ajani , et al. 2013 A trial of imaging selection and endovascular treatment for ischemic stroke. N. Engl. J. Med. 368:914–923. 23394476 10.1056/NEJMoa1212793 PMC3690785

[brb3455-bib-0023] Kolominsky‐Rabas, P. L. , P. U. Heuschmann , D. Marschall , M. Emmert , N. Baltzer , B. Neundörfer , et al. 2006 Lifetime cost of ischemic stroke in Germany: results and national projections from a population‐based stroke registry: the Erlangen Stroke Project. Stroke 37:1179–1183. 16574918 10.1161/01.STR.0000217450.21310.90

[brb3455-bib-0024] Lawrence, M. , S. Kerr , H. Watson , G. Paton , and G. Ellis . 2010 An exploration of lifestyle beliefs and lifestyle behaviour following stroke: findings from a focus group study of patients and family members. BMC Fam. Pract. 11:97. 21143874 10.1186/1471-2296-11-97 PMC3018456

[brb3455-bib-0025] Leistner, S. , S. Benik , I. Laumeier , A. Ziegler , G. Nieweler , C. H. Nolte , et al. 2012 Secondary prevention after minor stroke and TIA – usual care and development of a support program. PLoS One 7:e49985. 23284630 10.1371/journal.pone.0049985 PMC3524242

[brb3455-bib-0026] McAlister, F. A. , S. R. Majumdar , R. S. Padwal , M. Fradette , A. Thompson , B. Buck , et al. 2014 Case management for blood pressure and lipid level control after minor stroke: PREVENTION randomized controlled trial. CMAJ 186:577–584. 24733770 10.1503/cmaj.140053 PMC4016053

[brb3455-bib-0027] Mohan, K. M. , C. D. Wolfe , A. G. Rudd , P. U. Heuschmann , P. L. Kolominsky‐Rabas , and A. P. Grieve . 2011 Risk and cumulative risk of stroke recurrence: a systematic review and meta‐analysis. Stroke 42:1489–1494. 21454819 10.1161/STROKEAHA.110.602615

[brb3455-bib-0028] Philp, I. , M. Brainin , M. F. Walker , A. B. Ward , P. Gillard , A. L. Shields , et al. 2013 Development of a poststroke checklist to standardize follow‐up care for stroke survivors. J. Stroke Cerebrovasc. Dis. 22:e173–e180. 23265778 10.1016/j.jstrokecerebrovasdis.2012.10.016

[brb3455-bib-0029] Rabin, R. , and F. de Charro . 2001 EQ‐5D: a measure of health status from the EuroQol Group. Ann. Med. 33:337–343. 11491192 10.3109/07853890109002087

[brb3455-bib-0030] Ringelstein, E. B. , A. Chamorro , M. Kaste , P. Langhorne , D. Leys , P. Lyrer , et al. 2013 European Stroke Organisation recommendations to establish a stroke unit and stroke center. Stroke 44:828–840. 23362084 10.1161/STROKEAHA.112.670430

[brb3455-bib-0031] Röther, J. , M. J. Alberts , E. Touzé , J. L. Mas , M. D. Hill , P. Michel , et al. 2008 Risk factor profile and management of cerebrovascular patients in the REACH Registry. Cerebrovasc. Dis. 25:366–374. 18337635 10.1159/000120687

[brb3455-bib-0032] Schmidt, J. , F. Lamprecht , and W. W. Wittmann . 1989 Satisfaction with inpatient management. Development of a questionnaire and initial validity studies. Psychother. Psychosom. Med. Psychol. 39:248–255. 2762479

[brb3455-bib-0033] The National Institute of Neurological Disorders and Stroke rt‐PA Stroke Study Group . 1995 Tissue plasminogen activator for acute ischemic stroke. N. Engl. J. Med. 333:1581–1587. 7477192 10.1056/NEJM199512143332401

[brb3455-bib-0034] Vahedi, K. , J. Hofmeijer , E. Juettler , E. Vicaut , B. George , A. Algra , et al. 2007 Early decompressive surgery in malignant infarction of the middle cerebral artery: a pooled analysis of three randomised controlled trials. Lancet Neurol. 6:215–222. 17303527 10.1016/S1474-4422(07)70036-4

[brb3455-bib-0035] Ward, A. B. , C. Chen , B. Norrving , P. Gillard , M. F. Walker , S. Blackburn , et al. 2014 Evaluation of the post stroke checklist: a pilot study in the United Kingdom and Singapore. Int. J. Stroke 9(Suppl. A100):76–84. 25088427 10.1111/ijs.12291

